# Procoagulant Phosphatidylserine-Exposing Platelets *in vitro* and *in vivo*

**DOI:** 10.3389/fcvm.2020.00015

**Published:** 2020-03-03

**Authors:** Emily C. Reddy, Margaret L. Rand

**Affiliations:** ^1^Developmental and Stem Cell Biology, Research Institute, The Hospital for Sick Children, Toronto, ON, Canada; ^2^Division of Haematology/Oncology, Translational Medicine, Research Institute, The Hospital for Sick Children, Toronto, ON, Canada; ^3^Departments of Laboratory Medicine & Pathobiology, Biochemistry, and Paediatrics, University of Toronto, Toronto, ON, Canada

**Keywords:** platelets, procoagulant, phosphatidylserine (PS) exposure, platelet activation, platelet apoptosis, hemostasis, thrombosis

## Abstract

The physiological heterogeneity of platelets leads to diverse responses and the formation of discrete subpopulations upon platelet stimulation. Procoagulant platelets are an example of such subpopulations, a key characteristic of which is exposure either of the anionic aminophospholipid phosphatidylserine (PS) or of tissue factor on the activated platelet surface. This review focuses on the former, in which PS exposure on a subpopulation of platelets facilitates assembly of the intrinsic tenase and prothrombinase complexes, thereby accelerating thrombin generation on the activated platelet surface, contributing importantly to the hemostatic process. Mechanisms involved in platelet PS exposure, and accompanying events, induced by physiologically relevant agonists are considered then contrasted with PS exposure resulting from intrinsic pathway-mediated apoptosis in platelets. Pathologies of PS exposure, both inherited and acquired, are described. A consideration of platelet PS exposure as an antithrombotic target concludes the review.

## Introduction

Blood vessel wall injury sets into play processes that lead to the formation of a hemostatic plug that stops the bleeding from the injury site. In primary hemostasis, platelets adhere to exposed subendothelium, resulting in their activation and aggregation, forming a platelet plug. Secondary hemostasis is initiated by tissue factor exposure at the site of vessel wall damage, resulting in formation, *via* the coagulation pathway, of covalently cross-linked fibrin that binds to, and stabilizes, the platelet plug (1).

It has long been recognized that activated platelets contribute in a major way to fibrin formation; this is well-exemplified in the cell-based model of coagulation (2). This procoagulant property of platelets, earlier termed platelet factor 3 availability (PF3a) and assayed by measuring the ability of platelets to promote thrombin and fibrin formation (3), results from exposure of the anionic aminophospholipid phosphatidylserine (PS) on the surface of activated platelets (4). PS, translocated from the internal to the external platelet membrane leaflet, facilitates the assembly of the intrinsic tenase complex [factor (F)VIIIa; FIXa; FX] and prothrombinase complex (FVa; FXa; prothrombin), contributing to the burst of thrombin generation in the propagation phase of coagulation. Specifically, the negatively charged γ-carboxyglutamate (Gla) residues at the NH_2_ termini of the vitamin K-dependent factors, FIX(a), FX(a), and prothrombin, interact with negatively charged PS *via* Ca^2+^. FVIII binds to PS *via* its C2 domain and FVa *via* its C1 and C2 domains (5, 6). Tenase and prothrombinase activities are enhanced by PS-containing membranes by up to three orders of magnitude (7–9). Phosphatidylethanolamine (PE), that also becomes exposed on the surface of activated platelets, can contribute to the enhanced thrombin formation (7, 10); the fatty acid chain length of PE, but not PS, regulates the ability to support coagulation, with platelet-specific PEs demonstrating optimum activity (11). Oxidized PE, specifically 12-hydroxyeicosatetraenoic acid (HETE)-PE, formed by activated platelets, is even more potent than native PE in enhancing thrombin generation (12).

It is recognized that tissue factor-expressing platelets also comprise a subpopulation of procoagulant platelets. However, a discussion of this type of procoagulant platelet is beyond the scope of this mini-review, and the reader is referred to several recent, relevant publications on the topic (13–18).

### Procoagulant Phosphatidylserine-Exposing Platelet Subpopulations and Nomenclature

A unique feature of procoagulant platelet formation is that only a subpopulation of activated platelets exposes PS. This was recognized over 25 years ago by flow cytometry (19) using fluorescently labeled annexin A5 that binds PS with high affinity in a Ca^2+^-dependent manner. Fluorescently labeled lactadherin that does not require Ca^2+^ is also used to detect PS-exposing platelets [e.g., Dasgupta et al. (20)]. Flow cytometric and microscopy studies have shown colocalization of FVIII(a), FIX(a), FX(a), FV(a), and prothrombin with PS-exposing platelets, confirming that these platelets serve as assembly sites for the intrinsic tenase and prothrombinase complexes (21–25).

Procoagulant platelet subpopulations have been referred to by a myriad of names in the literature; however, it is recognized that these platelets share the key characteristic of PS exposure. An early description of a subpopulation of PS-exposing platelets was as COAT (COllagen And Thrombin)-FV platelets formed in response to dual agonist activation. These platelets were characterized by high levels of FV on their surface, in addition to PS (22, 26). COAT-FV was later abbreviated to COAT when it was demonstrated that these platelets are also coated with fibrinogen, fibronectin, von Willebrand factor (VWF), and thrombospondin, among many other α-granule proteins, on their surface (27). Subsequently, this subpopulation has been termed coated platelets, denoting the coating of the platelets with procoagulant proteins, including fibrin (28–30). The distinct morphology of procoagulant platelets has led to the terminology of blebbing, balloon(ing), or balloon-like platelets (31–35). Procoagulant platelets have also been referred to as SCIP (sustained calcium-induced platelet morphology) platelets (36), necrotic/4-[N-(S-glutathionylacetyl)amino]phenylarsonous acid (GSAO)-binding platelets (37–39), superactivated platelets (40), capped platelets (21, 41), and zombie platelets (42). Agbani and Poole (43) recently proposed “procoagulant platelets” as the unifying term for this activated platelet subpopulation.

In this brief review, we focus on mechanisms involved in PS exposure induced by platelet activation to form a subpopulation of procoagulant platelets, then contrasting it with PS exposure resulting from platelet apoptosis. Pathologies of PS exposure, inherited and acquired, are described. We conclude with a consideration of platelet PS exposure as an antithrombotic target.

## Platelet Membrane Phospholipid Asymmetry: Maintenance and Collapse

### Flippase [(Aminophospholipid) Translocase]

Similar to other biological membranes, resting platelets possess an asymmetrical phospholipid plasma membrane bilayer (4), with the minor phospholipid PS sequestered to the inner cytoplasmic leaflet. This PS asymmetry is created by a flippase/(amino)phospholipid translocase enzyme, a member of the Type IV subfamily of P-type ATPases (P4-ATPases) (9, 44), that rapidly and specifically shuttles PS from the outer to the inner membrane leaflet against the concentration gradient, in an ATP-dependent fashion (45). Its activity is abrogated when cytoplasmic Ca^2+^ (Ca^2+^_cyt_) increases to low micromolar levels (46).

### Scramblase and TMEM16F

Scramblase is a Ca^2+^-dependent, ATP-independent enzyme that regulates the rapid, non-specific bidirectional movement, i.e., “scrambling,” of phospholipids between membrane leaflets, resulting in a loss of normal membrane phospholipid bilayer asymmetry. Although scramblase activity has long been described in platelets (4), the protein involved in Ca^2+^-dependent PS exposure was only identified a decade ago as TMEM16F (anoctamin 6) (47–49). It is a member of the multiple transmembrane (TMEM)16 (anoctamin) domain family of proteins, of which the first-described member, TMEM16A, is a Ca^2+^-activated Cl^−^ channel. TMEM16F has been described to be a Ca^2+^-dependent Cl^−^ channel, a Ca^2+^-regulated non-selective cation channel permeable for Ca^2+^, or a Ca^2+^-dependent phospholipid scramblase (49). Evidence is accumulating that TMEM16F is indeed itself a scramblase [e.g., Watanabe et al. (50); Le et al. (51)].

## Platelet Phosphatidylserine Exposure

Several different pathways result in procoagulant platelet formation. In one, PS exposure occurs rapidly *via* platelet activation by strong agonists. A second is the intrinsic apoptosis pathway *via* which PS exposure occurs more slowly (9, 37, 52, 53). These pathways are considered in turn below, and key characteristics are summarized in Table 1. In a recently described third pathway that is distinct from the aforementioned canonical pathways, binding of oxidized low-density lipoprotein to platelet membrane glycoprotein (GP)IV (CD36) and signaling through extracellular signal-regulated protein kinase (ERK)5 mitogen-activated protein (MAP) kinase leads to PS exposure. This pathway may be relevant in thrombotic events that occur in dyslipidemia (54).

**Table 1:** Summary of key characteristics of platelet agonist- and apoptosis-induced PS exposure (Agonist-Induced Phosphatidylserine Exposure and Apoptosis-Induced Phosphatidylserine Exposure).

	**Agonist-induced platelet PS exposure** **(fast response)**	**Apoptosis-induced platelet PS exposure** **Dependence on MPTP (slow response)**
Trigger	*Physiologically relevant:* engagement of both GPVI (collagen/convulxin/CRP) and PAR1/PAR4 (thrombin) receptors *Non-physiological:* Ca^2+^ ionophores A23187, ionomycin	Inhibition of pro-survival Bcl-xL, resulting in activation of proapoptotic Bak and Bax *Non-physiological*: ABT-737
Cytoplasmic Ca^2+^ (Ca^2+^_cyt_) concentrations	Sustained, elevated Ca^2+^_cyt_ levels required	Not dependent on sustained, elevated Ca^2+^_cyt_ levels
Mitochondrial integrity	Loss of IMM integrity: • Dependence on MPTP formation • ΔΨ_m_ depolarization occurs	MOMP occurs early, with loss of IMM integrity occurring concomitant with PS exposure
Intracellular protease activation	• Calpain activated • Caspase activation occurs, but PS exposure is not dependent on it	• Dependent on caspase activation
TMEM16F	Required	Not essential
Morphology	• Rounded, blebbing platelets essentially empty of cytoplasmic contents • EVs are shed	• Rounded, blebbing platelets with cytoplasmic contents remaining • EVs are shed
*In vivo* effects (in animal models)	• In arterial thrombi, PS-exposing platelets form microdomains that do not participate in platelet aggregation • PS-exposing platelets continue to circulate in the bloodstream	• Formation of venous thrombi inhibited • Thrombocytopenia, with PS exposure persisting on remaining circulating platelets

*Ca^2+^_cyt_, cytoplasmic Ca^2+^; CRP, collagen-related peptide; EV, extracellular vesicle; GP, glycoprotein; IMM, inner mitochondrial membrane; MOMP, mitochondrial outer membrane permeabilization; MPTP, mitochondrial permeability transition pore; PAR, protease-activated receptor; PS, phosphatidylserine; ΔΨ_m_, inner mitochondrial membrane potential*.

### Agonist-Induced Phosphatidylserine Exposure

Agonist-stimulated platelet surface PS exposure is a rapid process, occurring in seconds to minutes, and is accompanied by other apoptotic-like events, including mitochondrial membrane permeabilization and depolarization, and plasma membrane blebbing, with extracellular vesicle (EV) formation. The proportion of PS-exposing platelets formed depends on the agonist(s) used for platelet stimulation, with the most potent *in vitro*, physiologically relevant stimulus being the combination of collagen/convulxin/collagen-related peptide (CRP) plus thrombin (C+T) The former binds to GPVI, and the latter cleaves protease-activated receptor (PAR)1 and PAR4 (23), synergizing to set into motion the signaling pathways that result in the sustained, supramaximal levels of Ca^2+^_cyt_ (55) (see below) that are required for PS exposure on a substantial proportion of platelets. Anywhere from 20 to 40% of C+T-stimulated platelets become PS-exposing, with a wide variation between donors; singly, these agonists are not as potent, with a smaller proportion of PS-exposing platelets being formed (56). ADP or thromboxane A_2_ (TxA_2_) (using the stable mimetic U46619) does not play a major role (23, 33), while shear forces are effective (57, 58). The non-physiological, non-receptor-mediated ionophores A23187, and ionomycin, that directly increase Ca^2+^_cyt_, are the most potent stimulators of PS exposure, with typically >90% of platelets taking on the procoagulant phenotype (19, 56).

Platelet stimulation by collagen (or convulxin/CRP) or thrombin [or the PAR-specific thrombin receptor activating peptides (TRAPs)] alone activates phospholipase (PL)Cγ (*via* GPVI) and PLCβ (*via* PAR1/4), resulting in a rise in Ca^2+^_cyt_ to the micromolar range (23). Cleavage of membrane phosphatidylinositol-4,5-bisphosphate by PLC forms inositol trisphosphate (IP_3_) and diacylglycerol (DAG); the former induces the release of Ca^2+^ from internal stores, the dense tubular system (DTS), *via* IP_3_ receptors. Depletion of internal Ca^2+^ stores allows store-operated Ca^2+^ entry (SOCE) from the platelet exterior: briefly, stromal interaction molecule 1 (STIM1) in the DTS membrane undergoes a conformational change, allowing activation of Orai1, the major Ca^2+^ release-activated Ca^2+^ channel in the platelet plasma membrane (23). DAG, together with Ca^2+^, activates protein kinase C (PKC)α that enhances Na^+^/Ca^2+^ exchange during SOCE (59, 60). Sustained increases in Ca^2+^_cyt_ *via* release from internal stores, SOCE, and release of mitochondrial Ca^2+^ upon mitochondrial permeability transition pore (MPTP) formation (see below) in a small proportion of platelets activate scramblase. It is the dual stimulation of GPVI and PAR1/4 by C+T that leads to the sustained, elevated levels of Ca^2+^_cyt_ necessary for PS exposure in a substantial proportion of platelets; the combination of C+T activates store-independent, receptor-operated Ca^2+^ entry (ROCE). This involves non-selective cation transient receptor potential C (TRPC) channels, TRPC3 and TRPC6, that allow Na^+^ entry. Coupling to reverse-mode Na^+^/Ca^2+^ exchange then leads to the sustained elevated Ca^2+^_cyt_ that activates scramblase (61).

Ca^2+^_cyt_ increases activation of the Ca^2+^-dependent cysteine protease calpain that has a number of substrates in platelets, including cytoskeletal components, signaling molecules, and the β3 integrin subunit, thereby regulating many platelet responses, including spreading, secretion, aggregation, and EV formation (36, 62–64). In platelets stimulated to expose PS, calpain-2-mediated proteolysis of αIIbβ3-associated proteins and β3 results in inactivation of αIIbβ3, the integrin necessary for platelet aggregation (65); thus, procoagulant platelets are unable to participate in aggregation. To that end, both in flow chambers coated with collagen and in mouse models of arterial thrombosis, two distinct microdomains of platelets are visualized in thrombi: 1) aggregated, non-PS-exposing platelets with extended pseudopods and activated αIIbβ3; surrounded by 2) PS-exposing platelets that have elevated Ca^2+^_cyt_, inactivated αIIbβ3, a rounded morphology, and are shedding EVs (see below) (32, 36). Further, in these *ex vivo* and *in vivo* systems, PS-exposing platelets are observed to translocate to the surface of thrombi where they accelerate fibrin formation (66).

Mitochondrial integrity loss is an apoptosis hallmark that precedes agonist-induced PS exposure, with involvement of the inner mitochondrial membrane (IMM) Formation of the cyclophilin D-regulated MPTP, a non-selective multiprotein pore that spans the IMM, is a key step (67, 68), as PS exposure is reduced in convulxin+thrombin-stimulated cyclophilin D-deficient platelets or platelets treated with cyclosporin A, an MPTP inhibitor (69–71). Reactive oxygen species, e.g., hydrogen peroxide (H_2_O_2_), that can trigger MPTP formation, synergize with thrombin to expose platelet PS, indicating a role for oxidative stress in procoagulant platelet formation (69). Sustained MPTP formation leads to disruption of the IMM potential (ΔΨ_m_) (72, 73), and ΔΨ_m_ depolarization is associated with PS exposure both in agonist-stimulated platelets *in vitro* and platelets aging *in vivo* (69, 71, 74–77).

In convulxin+thrombin-stimulated platelets, PS exposure is entirely dependent on ΔΨ_m_ loss and TMEM16F. However, there is evidence of a second minor pathway of PS exposure that occurs with collagen+thrombin stimulation that is independent of these (76, 78). This pathway may also be involved in the mitochondrial depolarization-independent PS exposure observed in A23187-stimulated platelets in the presence of cyclosporin A (79). Heterogeneity within the PS-exposing platelet subpopulation has also been reported by Topalov et al. (80): one subset with high Ca^2+^_cyt_, ΔΨ_m_ loss, and inactive αIIbβ3; and another with low Ca^2+^_cyt_, intact ΔΨ_m_, and active αIIbβ3. Subsequently, this latter subset was described to be the result of the interaction between a procoagulant platelet and an aggregatory (non-PS-exposing) platelet (81).

Although caspase activation occurs upon mitochondrial depolarization and has been used as a marker in studies of agonist-induced platelet PS exposure, e.g., caspase-3 (75, 79), the agonist-induced pathway of procoagulant platelet formation appears to be independent of caspase activation (52, 82).

Near-complete shedding of GPIbα and GPVI mediated by ADAM17 and 10, respectively, is accompanied by PS exposure and modulates platelet function from less adhesive to more procoagulant (83).

**Figure 1:**
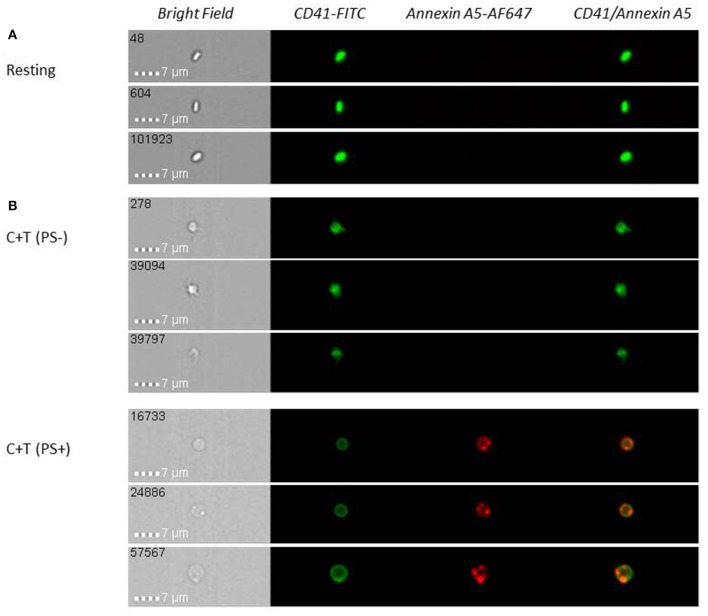
Upon platelet activation with collagen (10 μg/ml) + thrombin (1 U/ml) (C + T), a subpopulation of phosphatidylserine (PS)-exposing platelets with a round, balloon-like morphology and one or more associated extracellular vesicles (EVs) is formed. Resting (unstimulated) **(A)** or stimulated **(B)** washed platelets were labeled with anti-CD41-FITC (platelet marker) and annexin A5-Alexa Fluor 647 (PS exposure) and analyzed on an Amnis ImageStream^X^ Mark II (IS^X^) multispectral imaging flow cytometer. Images representative of *n* = 4 independent experiments.

In becoming procoagulant, platelets undergo remarkable morphological changes. Platelets adherent to collagen/CRP, but not fibrinogen, spread and transform into blebbing, rounded, balloon-like structures (31, 34). These collagen-adherent balloon platelets are PS exposing, as determined by annexin A5 binding (32, 33). Similarly, platelets stimulated in suspension by C+T or A23187 form a distinct PS-exposing platelet subpopulation with a spherical, balloon-like morphology, almost devoid of granules and normal internal architecture (Figure 1) (35, 84). This ballooning has been attributed to activation of Ca^2+^-activated Cl^−^ channels, resulting in initial salt entry into platelets, which is then followed by the influx of water (33). There is increased permeability of plasma membrane of the PS-exposing platelets to low-molecular-weight molecules (33, 71).

The unique surface protein coating of procoagulant platelets (Introduction) has been observed primarily localized to a small, convex structure, or cap, rather than distributed uniformly on the PS-exposing platelet surface; 85% of PS-exposing platelets possess caps, with one per platelet (21, 41). It was subsequently demonstrated that procoagulant activity is located in the cap, or remnant platelet body, early in balloon-platelet development, then becomes predominant in the balloon-like structure at later time points (85). Recently, it was observed that more than 90% of PS-exposing platelets possess one or multiple associated EVs that exhibit heterogeneity in platelet membrane glycoproteins and activation markers different from platelet-derived EVs free in suspension (see below) (Figure 1) (35).

PS exposure is accompanied by the release of membrane-bound EVs (previously referred to as microparticles or microvesicles) from the platelet plasma membrane (86); indeed, Scott syndrome platelets, that are deficient in PS exposure upon activation (Pathologies of Phosphatidylserine Exposure), are also deficient in EV formation (87, 88). EV surface membranes are heterogeneous in their expression of platelet membrane glycoproteins, e.g., αIIbβ3 and GPIb-IX-V, and activation markers, e.g., CD62P (P-selectin), CD63, and activated αIIbβ3 (35, 89, 90), and only about half expose PS (86). Platelet-derived EVs support hemostasis and also play a role in platelet–cell communications, delivering bioactive molecules, e.g., cytokines, eicosanoids, RNA species, to target cells [e.g., Boilard et al. (91)]. Elevated circulating EV levels have been reported in thrombotic conditions, immune-mediated conditions, and malignancy and inflammatory conditions, but whether they are “active contributors” or “passive indicators” of these conditions is not known (86). EVs are cleared rapidly (92), implying that they must be produced continuously for circulating levels to be detected.

Once procoagulant platelet formation has been stimulated *in vitro*, PS exposure is not readily reversed; this is likely due, at least in part, to inhibition of translocase activity (56, 76, 93), preventing the flipping of PS to the internal membrane leaflet. Even *in vivo*, PS exposure persists on activated platelets, as demonstrated by a study in which rabbit platelets stimulated to expose PS *in vitro* continued to circulate when injected into recipient rabbits (94). Although PS is a clearance signal of apoptotic cells by macrophages (see below), there are examples of cells that express PS constitutively and are viable (95, 96).

It is still not clear why there is platelet response heterogeneity to becoming PS exposing. Certainly, it is not due to differences in overall platelet reactivity, as PS-exposing platelets express CD62P to the same extent as non-PS-exposing platelets (9), indicating that they are capable of the secretion event. Although it had previously been attributed to platelet age, with young platelets, identified by increased thiazole orange staining, having an enhanced capacity to take on a procoagulant phenotype (22, 97), uptake of this dye is increased in large platelets that are not necessarily young platelets in the steady state (98). Recently, it has been shown that young, newly produced, steady-state (rabbit) platelets are indeed less responsive in exposing PS than older platelets (98). It is speculated that differences in receptor expression levels and in Ca^2+^-flux machinery activity are involved (99), and levels of adhesive receptors, including GPVI, GPIbα, αIIb, and β3, have been reported to be increased on procoagulant platelets (97, 100).

### Apoptosis-Induced Phosphatidylserine Exposure

It is now well-recognized that platelets, although they are anucleate, can undergo apoptosis. This occurs *via* the intrinsic, mitochondrial-dependent pathway, with platelets possessing the necessary cytosolic machinery, while likely lacking the death receptors required for the extrinsic pathway (37, 101, 102). Platelet apoptosis can be initiated *in vitro* by ABT-737, a BH3-only protein mimetic, that inhibits the pro-survival Bcl-2 family protein Bcl-xL, resulting in activation of the proapoptotic Bcl-2 proteins Bak and Bax; these then go on to initiate mitochondrial damage, cytochrome c release, caspase activation, PS exposure, and membrane blebbing and EV formation (37, 52, 101), all hallmarks of apoptosis in nucleated cells.

In contrast to platelet agonist-induced PS exposure, which occurs rapidly, apoptosis-induced PS exposure occurs more slowly, on the order of hours (9, 37, 52, 53). This apoptosis pathway is indeed Bak and Bax dependent, as platelets lacking these proteins do not externalize PS when incubated with ABT-737. It is also caspase dependent, as responses are abolished in the presence of a caspase inhibitor (52, 78). However, it does not require increases in Ca^2+^_cyt_ or calpain activity, which are necessary for agonist-induced PS exposure (52) (Agonist-Induced Phosphatidylserine Exposure) There is early mitochondrial outer membrane permeabilization (MOMP), followed by later IMM disruption concomitant with PS exposure (81): MPTP formation may not be involved, and ΔΨ_m_ depolarization may occur (52, 103–105). PS-exposing platelets take on a rounded morphology but maintain cytoplasmic components (105, 106); EV formation is not observed early on, but increases with time (103, 104, 107).

ABT-737 treatment of platelets has also been reported to result in PS exposure on a second platelet population at a higher level, observed with agonist-induced PS exposure, than described above. PS exposure on this population is dependent on increases in Ca^2+^_cyt_ and TMEM16F but unlike agonist-induced PS exposure is dependent on caspase activation (78, 107).

Apoptosis, being the process of programmed cell death, is a major physiological mechanism that regulates the life span of cells, with externalized PS being the “eat me” recognition signal for phagocytic cells to mediate clearance of damaged cells (108). Platelet apoptosis regulates circulating platelet life span, as mutations in Bcl-xL result in shortened platelet survival, while deletion of Bak and Bax prolongs it by almost 2-fold (109, 110). Administration of ABT-737 to dogs and mice causes dramatic thrombocytopenia within 2 h (109, 111), but PS exposure persists on the circulating platelets (106); formation of venous thrombi is inhibited (106). Platelet PS exposure may be involved in physiological platelet clearance; the proportion of PS-exposing platelets increases as rabbit platelets age in the circulation under steady-state conditions (77).

## Pathologies of Phosphatidylserine Exposure

The importance of activated platelet membrane phospholipid bilayer scrambling with resulting PS exposure in hemostasis is highlighted in the very rare inherited autosomal recessive disorder Scott syndrome. The first-described patient, Mrs. M.A. Scott, had a relatively severe bleeding phenotype: she was found to have an isolated defect in PF3a (Introduction) (112); impaired PS exposure upon platelet activation, thereby resulting in deficient procoagulant activity and abrogated fibrin formation at sites of vascular damage (3, 113); and diminished EV formation (Agonist-Induced Phosphatidylserine Exposure) The genetic defect in four of the six known Scott syndrome patients for whom mutational analysis is available, as well as in canine Scott syndrome, in German Shepherd dogs, involves homozygous and heterozygous variants in the *TMEM16F* gene (47, 49, 114–118), resulting in an absence of expression of the TMEM16F protein (Scramblase and TMEM16F) Knockout of *TMEM16F* in genetically modified mice recapitulates the Scott syndrome phenotype (119–122).

Studies of Scott syndrome platelets have shown that, in humans, TMEM16F is required for the major agonist-induced PS exposure pathway but is not essential for apoptosis-induced PS exposure (78). In contrast, in dogs, both agonist- and apoptosis-induced platelet PS exposure requires TMEM16F (116). Detailed proteomic profiling of human Scott syndrome platelets has provided insight into protein modifications that occur when platelets are activated to expose PS (123).

In contrast with the Scott syndrome, in the rare autosomal dominant Stormorken syndrome, resting platelets have elevated surface PS exposure; resting Ca^2+^_cyt_ is increased due to a novel *STIM1* gain-of-function variant. Stormorken syndrome patients have thrombocytopenia and a mild bleeding diathesis along with their thrombocytopathy (124).

Another inherited disorder in which elevated PS exposure and Ca^2+^_cyt_ is observed with resting platelets is the microthrombocytopenia, Wiskott–Aldrich syndrome (WAS) Upon stimulation, WAS platelets have increased susceptibility to PS exposure that occurs as a result of MPTP opening (125, 126).

Resting platelets from Bernard–Soulier syndrome patients also have elevated PS exposure, independent of their large size (127), indicating a role for GPIb-IX-V. PS exposure is generally increased in activated BSS platelets as well and is accompanied by ΔΨ_m_ depolarization in a proportion of platelets (127).

Aberrant PS exposure has been described in certain acquired platelet disorders. Platelets from patients with immune thrombocytopenia have increased (apoptosis-induced) PS exposure likely contributing to the decreased platelet counts (128, 129). Recently, there has been the report of increased circulating PS-exposing ballooned platelets in trauma hemorrhage in response to the damage-associated molecular pattern histone H4, demonstrating a mechanism by which platelets respond to tissue damage (130).

## Conclusion: Potential of Procoagulant Phosphatidylserine-Exposing Platelets as an Antithrombotic Target

Understanding of the mechanisms involved in the formation of the procoagulant PS-exposing platelet phenotype and its role in hemostasis has increased dramatically. Since traditional antiplatelet agents do not completely reduce the risk of thromboembolic events, the question arises: Is PS exposure a useful antithrombotic target? Not surprisingly, drugs that target the ADP and TxA_2_ pathways of platelet activation and aggregation have no major effect on inhibiting platelet procoagulant activity (38, 131, 132) since these agonists are not particularly potent in stimulating the formation of PS-exposing platelets (Agonist-Induced Phosphatidylserine Exposure). There are certainly indications that the procoagulant platelet might be a useful target in reducing thrombosis. Firstly, knockout of *TMEM16F* in platelets of genetically modified mice (Pathologies of Phosphatidylserine Exposure) decreases platelet thrombus formation *in vitro* on collagen-coated coverslips under flow conditions and in models of arterial and venous thrombosis (119–122). Secondly, there is evidence that in clinical conditions of thrombosis, specifically coronary artery disease, and essential thrombocythemia, the procoagulant platelet response is increased, and that increased levels of procoagulant platelets are associated with increased risk for recurrent infarction in lacunar and non-lacunar stroke and predict incident stroke after transient ischemic attack (131, 133–136). Thirdly, procoagulant platelets have recently been shown to play a critical role in forming neutrophil macroaggregates that promote pulmonary thrombosis after gut ischemia that is a potent inducer of platelet PS exposure on the endothelium in the intestines, liver, and lungs; large membrane fragments ripped from PS-exposing platelets in a shear-dependent fashion wrap around the neutrophils to form adhesive bridges (137).

Thus, since PS exposure persists on activated platelets not only *in vitro* but *in vivo* as well (76, 94), blocking of the procoagulant surface could be an effective, novel strategy to reduce thrombosis. By binding to PS, annexin A5, diannexin [a recombinant annexin A5 homodimer with a longer circulating half-life than annexin A5 and a 10-fold higher binding affinity to PS (138, 139)] and lactadherin inhibit thrombus formation *in vitro* and in animal models of arterial and venous thrombosis (139–145). However, diannexin and lactadherin impair hemostasis as well, increasing murine tail bleeding time blood loss (139, 145); thus, if a strategy of blocking exposed PS is to be pursued, dosages of blocking compounds must be finely tuned.

Alternatively, mitochondrial depolarization (70), scramblase activity (146, 147), or water entry into platelets (33, 148) are potential targets to inhibit formation of the thrombin-generating subpopulation of platelets while still allowing platelet aggregation to occur. It may be that inhibition of procoagulant platelet formation could be an alternative approach to reduce thrombosis without impairing hemostasis.

## Author Contributions

All authors listed have made a substantial, direct and intellectual contribution to the work, and approved it for publication.

### Conflict of Interest

The authors declare that the research was conducted in the absence of any commercial or financial relationships that could be construed as a potential conflict of interest.
